# Single-stage total knee arthroplasty revision with extensor mechanism allograft: surgical technique

**DOI:** 10.1051/sicotj/2026014

**Published:** 2026-05-12

**Authors:** Yasmine Salese, Gerald Delfosse, Cécile Batailler, Elvire Servien, Sébastien Lustig

**Affiliations:** 1 Orthopaedics Surgery and Sports Medicine Department, FIFA Medical Center of Excellence, Croix-Rousse Hospital, Lyon North, University Hospital 69004 Lyon France; 2 Univ Lyon, Claude Bernard Lyon 1 University, IFSTTAR, LBMC UMR_T9406 69622 Lyon France; 3 LIBM – EA 7424, Interuniversity Laboratory of Human Movement Science Université Lyon 1 69008 Lyon France

**Keywords:** Extensor mechanism, Allograft, Revision total knee arthroplasty, Surgical technique

## Abstract

*Introduction*: Chronic rupture of the extensor mechanism is a serious complication that could occur in the context of revision total knee arthroplasty (TKA). Performing combined extensor mechanism allograft reconstruction and revision TKA as a single-stage procedure requires precise surgical technique. *Technique:* Allograft size and quality were assessed preoperatively. A tibial tubercle osteotomy (TTO) was performed prior to revision TKA. After implantation of the definitive prosthesis, the TTO was fixed to the tibia using screws. The quadriceps tendon was sutured to the allograft using the Pulvertaft weave technique with the knee in full extension. Native patellar retinacula and prepatellar fascia were preserved to optimize graft coverage. Postoperatively, patients were immobilized in full extension for three months before starting progressive mobilization. *Results:* From January 2017 to April 2024, 20 patients underwent a single-stage revision TKA with total extensor mechanism allograft and were followed for a minimum of one year. Range of motion was recovered at follow-up. Five patients (25%) had failures attributed to the allograft. *Conclusion:* Single-stage TKA revision combined with extensor mechanism allograft, in the context of multiply operated knees, requires meticulous stepwise execution, including secure tibial fixation, precise graft tensioning, and preservation of native soft tissue coverage, to optimize outcomes in this high-risk setting.

## Introduction

Chronic rupture of the extensor mechanism is a rare but serious complication following total knee arthroplasty (TKA) [1]. Surgical management options include reconstruction with various types of allografts (i.e., Achilles tendon grafts, partial or total extensor mechanism allografts [2–5]) or, as a last resort, knee arthrodesis. Among these approaches, total extensor mechanism allografts have demonstrated promising long-term outcomes [4, 6, 7]. If rupture is associated with instability, stiffness, or implant loosening, or if it follows septic complications, the reconstruction may be performed concurrently with a TKA revision (RTKA). Combining these procedures increases the complexity and duration of the operation and exposes the patient to several complications (allograft failure, stiffness, infection). Fixing the anterior tibial tuberosity in the context of tibial implant revision surgery requires a skilled surgeon. This article describes the surgical technique and clinical outcomes of total extensor mechanism allograft reconstruction performed in a single stage with RTKA.

## Material and methods

This single-center retrospective study analyzed patients who underwent surgery between January 2017 and April 2024.

Indications included chronic or recurrent extensor mechanism rupture, a history of total patellectomy, a complete or ≥ 30° active extension deficit (AED), in the presence of an indication for RTKA (Table 1). Contraindications included uncontrolled infection, inadequate soft tissue coverage, acute extensor mechanism rupture without prior repair attempts or autograft reconstruction, and patients with low functional demand.

**Table 1:** Etiologies of extensor mechanism failure and indications for RTKA.

Patient (n°)	Extensor mechanism lesion	TKA issue	Active extension deficit (AED)
1	Allografted PT rupture	Tibia aseptic loosening	0°
2	Allografted PT rupture	Iterative septic loosening	0°
3	PT rupture	Femoropatellar prothesis	30°
4	PT rupture	Iterative septic loosening	0°
5	Patellectomy	Septic loosening	60°
6	PT rupture	Femoral implant malposition	30°
7	Patellectomy	Iterative septic loosening	30°
8	Allografted AT rupture	Iterative septic loosening	20°
9	QT rupture	Aseptic loosening	30°
10	QT rupture	Recurvatum	20°
11	PT rupture	Septic loosening, TTO	50°
12	Patellar disclocation fracture	Iterative septic loosening	Complete AED
13	PT rupture, ATT fracture, open patellar luxation	Iterative septic loosening	50°
14	QT rupture	Iterative aseptic loosening	Complete AED
15	Patellectomy	Instability	20°
16	PT rupture	Instability	0°
17	PT rupture	Septic loosening	40°
18	ATT fracture	Iterative septic loosening	0°
19	PT rupture	Iterative septic loosening	20°
20	QT rupture	Iterative aseptic loosening	0°

AED: active extension deficit, PT: patellar tendon, QT: quadricipital tendon, ATT: anterior tibial tuberosity, TTO: tibia tubercle osteotomy.

During this period, a final cohort of 20 patients met the inclusion criteria. The surgical technique was identical for every case. Follow-up was recorded, and postoperative complications were categorized into three groups: those related to RTKA, allograft, or infection. Failure was defined as an AED of ≥ 30°, revision surgery for allograft failure, revision for infection (excluding debridement, antibiotics, and implant retention [DAIR]), or revision for TKA loosening. Statistical analysis was conducted using XLSTAT software. Survival rates were estimated using the Kaplan–Meier method with 95% confidence intervals.

## Surgical technique

The entire surgery is available on Video 1.

### Preoperative planning

frozen extensor mechanism allografts were preferred for their superior biological properties, and fresh-frozen grafts were favored over irradiated ones [8]. The graft dimensions were appropriate to restore patellar thickness, width, and height, as well as the anatomical dimensions of the tendons, which were evaluated using radiographs of the contralateral knee. Particular attention was paid to the dimensions of the tibial tubercle osteotomy (TTO) and the length of the quadriceps tendon (QT). After thawing and before skin incision, the graft was inspected, measured, and a systematic bacteriological sample was taken.

### Surgical approach

The patient was positioned supine, with the operative leg draped in the field, supported by a foot support and a lateral support, without a tourniquet. In patients with multiple prior incisions, the most lateral one was reopened. Arthrotomy was performed through the area of the extensor mechanism rupture. A patellectomy was carried out. If the native patellar tendon was still present, it was split longitudinally up to the anterior tibial tuberosity. The native patellar retinaculum and prepatellar fascia were preserved to enhance graft coverage. The previous TKA components were carefully removed using saws and chisels to minimize bone loss. Residual cement and any intramedullary plugs were also removed.

### Allograft preparation

**Figure 1:**
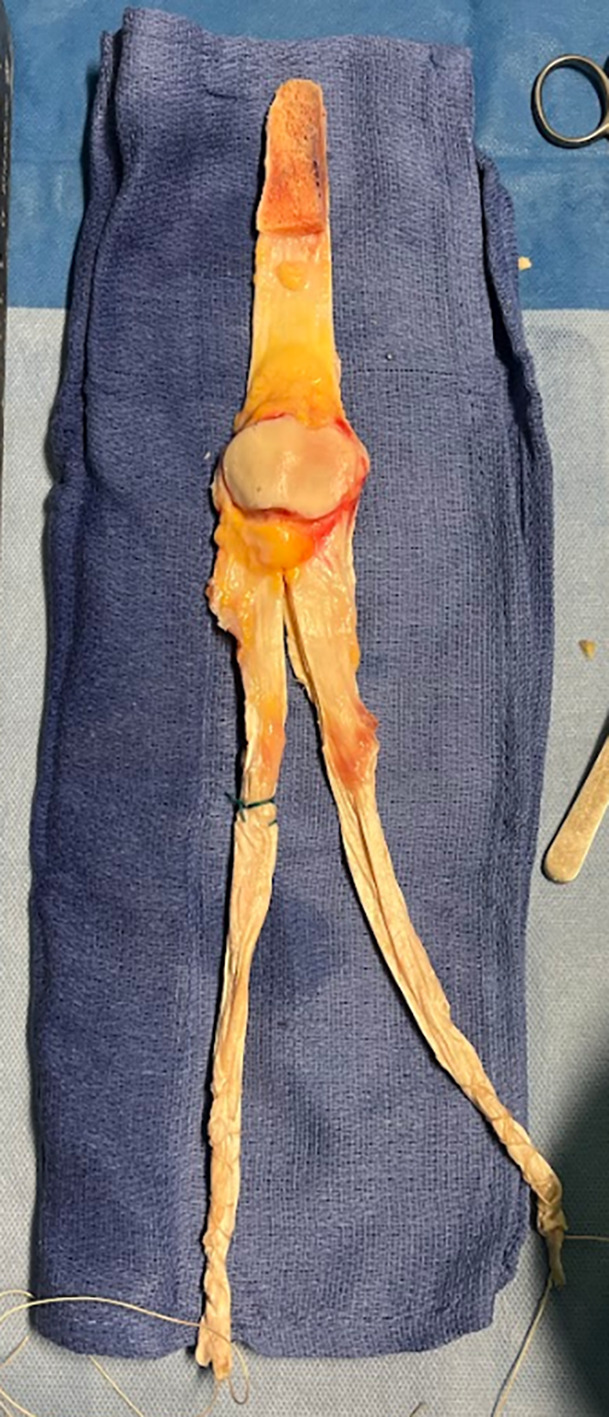
Allograft preparation.

The TTO of the allograft was shaped into a bone block of approximately 6 × 1.5 × 2 cm [9] (Figure 1). The QT was split into two bundles, using traction sutures [10].

## Reimplantation

**Figure 2:**
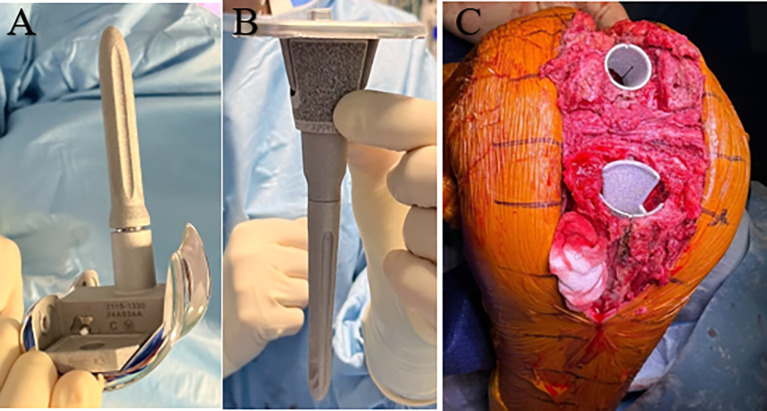
Example of RTKA: semi-constrained prosthesis with distal wedges, stems, and tibial cone (A, B), tibial and femoral cones in situ (C).

The first step following removal of the previous TKA components was evaluation of bone loss prior to new femoral and tibial bone cuts [11]. The second step was then the revision of bone cuts. The third step was the choice of implants and level of constraint, tailored to each case. In situations involving moderate to severe bone loss, metaphyseal cones were used to enhance fixation. The use of metallic wedges was based on the extent and location of bone deficiency (Figure 2). The fourth step consisted of trials positioning and coronal laxity in flexion and extension was tested.

Trials in place, the fifth step was preparation of the future TTO site, using saws and chisels to create a receiving socket matching the size of the allograft. The target dimensions were approximately 6 cm in length, 1.5 cm in depth, and 2 cm in width, with clean edges to allow press-fit fixation [9] (Video 2). Then trials were removed, and the sixth step consisted of cementation. All implants, except for cones, were cemented using antibiotic-loaded cement and diaphyseal plugs. Particular attention was paid to tibial osteotomy to prevent cement leakage.

### Allograft

**Figure 3:**
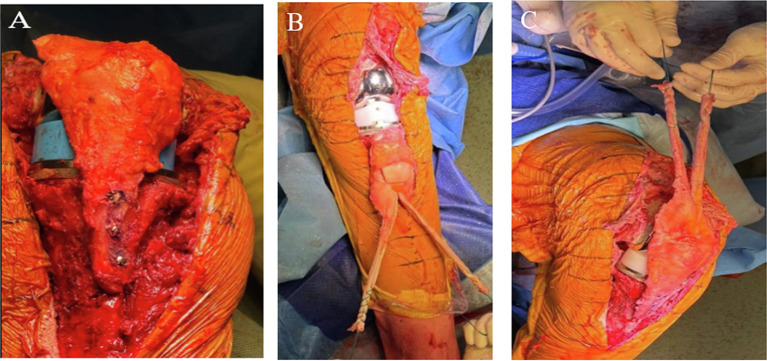
TTO fixation (A, B, C). A: anterior tibial tuberosity fixation. (B), (C): proximal fixation: the two bundles of the QT graft are passed through the native QT using three weaves per limb, according to the Pulvertaft technique. The graft was then secured with multiple non-absorbable sutures. During suturing, tension was applied in opposite directions on the allograft and native QT strands to ensure optimal tensioning of the reconstructed extensor mechanism.

Fixation of the allograft was performed with the knee in full extension. The graft was initially impacted for a press-fit and then fixed to the tibia using two to three screws (3.5 or 4.5 mm in diameter) [9] (Video 3). Metal washers might be added if necessary. For proximal fixation, the two bundles of the QT graft were passed through the native QT using three weaves per limb, according to the Pulvertaft technique. The graft was then secured with multiple non-absorbable sutures. During suturing, tension was applied in opposite directions on the allograft and native QT strands to ensure optimal tensioning of the reconstructed extensor mechanism (Figure 3). The native patellar retinacula, the prepatellar fascia, and, if present, the native patellar tendon were then sutured to the allograft. (Video 4). The patella of the allograft was not resurfaced to avoid the risk of graft osteolysis. Patellar tracking was assessed in slight flexion to prevent displacement of fixation screws or fracture of the tibial allograft bone block before graft sutures. Intraoperative fluoroscopy was used to check patellar height and screws positioning.

### Postoperative care

**Figure 4:**
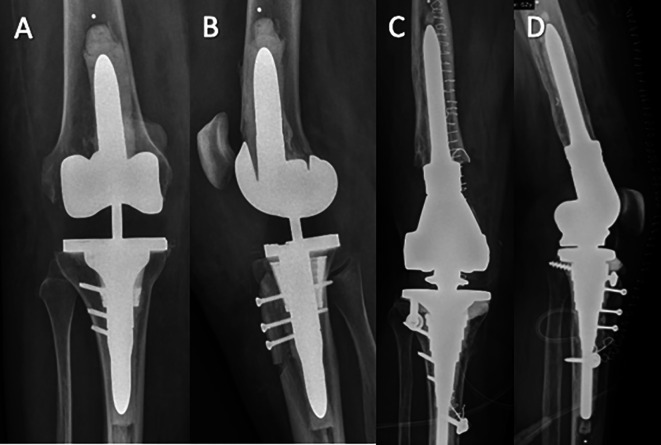
Postoperative radiographs: example of two different types of constraint. A, B: Varus Valgus Constraint implant; C, D: Rotating hinge. In both cases, TTO fixation was possible using screws despite tibial cone or sleeve.

Following radiological and clinical assessment, a knee brace in full extension was required for 3 months (Figure 4). Weight-bearing was permitted with the use of crutches and brace. Knee flexion was permitted in a gradual manner starting from the third postoperative month with 30-degree increments every 2 weeks. A hinged brace was maintained until full quadriceps locking was achieved. The rehabilitation protocol was supervised by a specialised physiotherapist for a minimum of six months to one year.

The minimum postoperative follow-up included radiological and clinical evaluations at 2, 3, and 6 months, with subsequent evaluations at 1 and 2 years post-surgery (Video 5).

## Results

Our cohort consisted of 20 patients, including 11 women (55%) and 9 men (45%), with a mean age of 65.3 years. As major risk factors, two patients (10%) were smokers, five patients (25%) had a BMI ≥ 30. The average follow-up duration was 31.6 months.

The cohort included 19 RTKA procedures (95%) and one revision of a femoro-patellar prosthesis (PFP) to a TKA (5%). Various implant types were used: 1 posterior-stabilized prosthesis (5%), 2 semi-constrained prostheses (10%), 15 rotating-hinge prostheses (75%), and 2 resection prostheses (10%).

Complications and etiologies of failure are listed in Table 2. Regarding postoperative complications, seven patients (35%) developed a total of eight complications related to RTKA. Five patients (25%) experienced rupture of the extensor mechanism allograft at different levels. Nine patients (45%) experienced septic complications. Fifteen patients (75%) underwent at least one additional surgical procedure due to complications. Functional flexion (>90°) was generally achieved between the fourth and sixth postoperative months. Mean flexion at last follow-up was 109° (70–130°).

**Table 2:** Results, complications, and failures.

Patient (n°)	Postoperative complications (related to RTKA, allograft or infection including DAIR)	Delay (months)	Failure (AED of ≥30°, revision for allograft failure, for infection excluding DAIR, for TKA loosening	Delay (months)	Postoperative ROM (°) (Recurvatum – Flessum – Flexion)
1	RTKA, allograft	32	Allograft and RTKA failure	32	0-0-90
2	0	0	0	0	0-0-130
3	Allograft	10	Allograft failure	10	0-0-70
4	Infection	1	0	0	5-0-90
5	RTKA, allograft	20	Allograft and RTKA failure	20	/ *
6	0	0	0	0	5-0-130
7	Infection	3	Infection	3	0-0-90
8	Infection	2	Infection	6	/ *
9	RTKA, allograft	18	AED of ≥30°, allograft and RTKA failure	18	0-0-130
10	Allograft	5	AED of ≥30°	5	0-0-110
11	Allograft	24	0	0	0-0-120
12	RTKA	9	AED of ≥30°	6	0-45-120
13	RTKA, infection	7	Infection	10	/ *
14	0	0	0	0	0-0-110
15	RTKA, allograft	23	RTKA failure	28	5-0-120
16	Infection	7	AED of ≥30°	13	0-0-90
17	Infection	1	AED of ≥30°, infection	2	0-20-120
18	Allograft, infection	4	Allograft failure, infection	4	/ *
19	Infection	1	0	0	0-0-120
20	RTKA	12	RTKA failure	24	0-0-95

* These patients underwent arthrodesis or transfemoral amputation. ROM: range of motion.

The survival rate without failure was 60% at 12 months. The mean survival time without failure was 18.35 months. The survival rate without allograft failure was 78.6% at 12 months. Infection-free survival rate (excluding DAIR cases) was 72.7% at 12 months and remained stable at the final follow-up.

## Discussion

The high complication and failure rates reflect the complexity of this highly selected cohort of multiply operated knees. In this setting, the procedure should not be regarded as a routine indication but rather as a salvage strategy prior to arthrodesis, definitive implant removal, or transfemoral amputation [12]. The goal is not to achieve optimal function, but to restore ambulation, active extension and flexion, and to reduce pain and disability.

No study has specifically evaluated total extensor mechanism allograft reconstruction combined with single-stage RTKA. Browne and Hanssen [13] reported a 38% failure rate in 50 reconstructions, with 56% 10-year failure-free survival. Complications included 8% graft rupture, 10% infection, and 20% extensor lag ≥30°. Ricciardi et al. [7] observed a 58% reoperation rate at 68 months in 26 knees, 19% of which were associated with RTKA.

Synthetic grafts appear to provide comparable results. Shau et al. [14], in a meta-analysis of 204 knees, found similar success rates for allografts (76%) and synthetic grafts (74%), without differences in complications. Synthetic grafts were more frequently used when revision TKA was required, likely because they allow simpler distal fixation. Achilles tendon allografts have shown comparable outcomes, with a 58.6% success rate at 42 months in 29 knees [15]. We nevertheless favor total extensor mechanism allografts for their more reliable proximal fixation and improved native tissue coverage.

All reconstruction techniques are associated with high rates of infection. The combination of RTKA and extensor mechanism reconstruction likely increases this risk due to prolonged operative time and soft tissue compromise.

This is, to our knowledge, the first study specifically addressing total extensor mechanism allografts combined with RTKA. Its limitations include a small sample size and a retrospective design, and the fact that subgroup analysis was not feasible.

## Conclusion

Total extensor mechanism allograft reconstruction combined with single-stage revision TKA is a demanding salvage procedure that requires meticulous stepwise surgical execution. Key technical aspects include accurate graft sizing, secure tibial tubercle fixation with press-fit and screw stabilization, precise proximal fixation using the Pulvertaft technique under appropriate tension, preservation of native soft tissue coverage, and strict postoperative immobilization.

When these technical principles are respected, this procedure provides a limb-preserving alternative to arthrodesis in complex, multiply operated knees. Despite the high complication rate inherent to this challenging population, it remains a valuable option in specialized referral centers, provided that patients are carefully selected and adequately informed of the surgery's salvage nature.

## Data Availability

Data generated and/or analyzed during this study are not publicly available due to legal and ethical restrictions related to patient confidentiality. All photographs and videos related to the surgical technique are available within the article and its supplementary material.

## Supplementary material

*Video 1*. Complete case surgery. *Video 2*. Preparation of tibial socket for allograft. *Video 3*. Knee testing after fixation of the allograft. *Video 4*. Native apparal suture on allograft in tension. *Video 5*. Walking outcome at six months. The supplementary material of this article is available at following https://www.sicot-j.org/10.1051/sicotj/2026014/olm.
